# Barriers and Facilitators for Referrals of Primary Care Patients to Blended Internet-Based Psychotherapy for Depression: Mixed Methods Study of General Practitioners’ Views

**DOI:** 10.2196/18642

**Published:** 2020-08-18

**Authors:** Ingrid Titzler, Matthias Berking, Sandra Schlicker, Heleen Riper, David Daniel Ebert

**Affiliations:** 1 Department of Clinical Psychology and Psychotherapy University of Erlangen-Nürnberg Erlangen Germany; 2 Faculty of Behavioral and Movement Sciences Section of Clinical Psychology VU University Amsterdam Amsterdam Netherlands; 3 Department of Research and Innovation GGZinGeest Amsterdam Netherlands

**Keywords:** barriers and facilitators, general practitioners, depression, referral, blended therapy, internet-based intervention, mobile phone, psychotherapy, qualitative research

## Abstract

**Background:**

Major depressive disorder (MDD) is highly prevalent and often managed by general practitioners (GPs). GPs mostly prescribe medication and show low referral rates to psychotherapy. Many patients remain untreated. Blended psychotherapy (bPT) combines internet-based interventions with face-to-face psychotherapy and could increase treatment access and availability. Effectively implementing bPT in routine care requires an understanding of professional users’ perspectives and behavior.

**Objective:**

This study aims to identify barriers and facilitators perceived by GPs in referring patients to bPT. Explanations for variations in referral rates were examined.

**Methods:**

Semistructured interviews were conducted with 12 of 110 GPs participating in a German randomized controlled trial (RCT) to investigate barriers to and facilitators for referrals to bPT for MDD (10 web-based modules, app-based assessments, and 6 face-to-face sessions). The interview guide was based on the theoretical domains framework. The interviews were audio recorded and transcribed verbatim, and the qualitative content was analyzed by 2 independent coders (intercoder agreement, k=0.71). A follow-up survey with 12 interviewed GPs enabled the validation of emergent themes. The differences in the barriers and facilitators identified between groups with different characteristics (eg, GPs with high or low referral rates) were described. Correlations between referrals and characteristics, self-rated competences, and experiences managing depression of the RCT-GPs (n=76) were conducted.

**Results:**

GPs referred few patients to bPT, although varied in their referral rates, and interviewees referred more than twice as many patients as RCT-GPs (interview-GPs: mean 6.34, SD 9.42; RCT-GPs: mean 2.65, SD 3.92). A negative correlation was found between GPs’ referrals and their self-rated pharmacotherapeutic competence, *r*(73)=−0.31, *P*<.001. The qualitative findings revealed a total of 19 barriers (B) and 29 facilitators (F), at the levels of GP (B=4 and F=11), patient (B=11 and F=9), GP practice (B=1 and F=3), and sociopolitical circumstances (B=3 and F=6). Key barriers stated by all interviewed GPs included “little knowledge about internet-based interventions” and “patients’ lack of familiarity with technology/internet/media” (number of statements, each k=22). Key facilitators were “perceived patient suitability, e.g. well-educated, young” (k=22) and “no conflict with GP’s role” (k=16). The follow-up survey showed a very high agreement rate of at least 75% for 71% (34/48) of the identified themes. Descriptive findings indicated differences between GPs with low and high referral rates in terms of which and how many barriers (low: mean 9.75, SD 1.83; high: mean 10.50, SD 2.38) and facilitators (low: mean 18.25, SD 4.13; high: mean 21.00; SD 3.92) they mentioned.

**Conclusions:**

This study provides insights into factors influencing GPs’ referrals to bPT as gatekeepers to depression care. Barriers and facilitators should be considered when designing implementation strategies to enhance referral rates. The findings should be interpreted with care because of the small and self-selected sample and low response rates.

## Introduction

### Background

Approximately 350 million people worldwide are affected by major depressive disorder (MDD) annually [1]. It is the most common mental health disorder, with an estimated lifetime prevalence of 12.8% [2]. Prevalence rates of MDD in primary care patients range from 10% to 14% [3-6].

General practitioners (GPs) are considered most important in the management of depression because they are often the first point of contact in the pathways to care [7,8]. Accordingly, it is estimated that 60% to 71% of patients with MDD are treated by their GP [5,9-11]. However, a recent study [12] showed that 60% of patients with MDD under primary care did not receive guideline-oriented treatment; 54% were treated by their GPs with medication only (31%), counseling (45%), referral to specialized care (21%), and psychotherapy (10%). In general, referral rates of GPs to specialized care for depression vary between 16% and 58% [3,13-16]. In Germany, psychotherapy referral rates are low (8%-33%) [13,17,18], and only 17.3% of patients with mild or moderate depression and 3.6% with severe depression are treated with psychological psychotherapy in a 3-year period [10].

There are several reasons for not adhering to clinical guidelines in primary care. Factors that may hinder GPs in adequately treating depression include low rates (47%) of correct recognition and diagnosis of depression [19], insufficient time for care provision [20], self-reported insufficient knowledge relating to diagnosis or treatment and low skills [16,20], and higher self-confidence in providing medication [4,16]. Studies have also identified factors influencing GPs’ referrals to mental health services [4,15,16,20-22]. These include characteristics of the disease (impairment, severity and duration of symptoms, and need for specialized treatment), patient (age, gender, and presentation of psychological complaints), GP (confidence in their abilities, insufficient time and insufficient skills to provide care, and need for clarification of diagnosis), and care system (lack of access to specialists, long distance or waiting time, personal communication, and medical exchange).

Some of the abovementioned barriers may be overcome by providing digital technologies as an additional treatment option. Digital approaches may help to improve access to specialists, decrease long waiting times, and reduce the high number of untreated patients [23,24]. Internet- and mobile-based interventions (IMIs) have been shown to be effective in treating MDD [25] and to yield results comparable with face-to-face psychological short-term treatments, when delivered as guided internet-based treatment [26].

Despite its potential, the uptake of evidence-based internet-delivered treatment remains limited [27,28], and not all patients are willing to use a digital stand-alone treatment [29,30]. For patients who are open to using technology-based treatments but also value personal contact, blended psychotherapy (bPT) might be a promising approach that combines IMIs with face-to-face treatment. This is a relatively new research field, and only a few studies have investigated the acceptance and effectiveness of blended treatments [31]. Previous studies indicate a potentially high acceptance and willingness to use bPT by both patients [32,33] and therapists [34-36]. Furthermore, randomized controlled trials (RCTs) on blended treatments indicated that the reduction of face-to-face psychotherapy sessions with equivalent replacement by IMIs did not lead to an inferior outcome when compared with traditional therapy [37-39]. Thus, implementing bPT could be an attractive alternative with the potential to reduce therapist time and increase the number of treated patients.

Given GPs’ central role as gatekeepers in the management of depression, research on their perspectives as gatekeepers seems crucial. A recent study on the implementation of internet-based therapy services in routine care, in guided, unguided, or blended formats, suggested that GPs’ low referral rates and unstable patient intake could be explained by GP skepticism and reservations and called for research on referrers’ attitudes [40]. A study on Australian routine practice not only indicated a high GP satisfaction with web-based referrals and treatment services but also highlighted challenges in encouraging GPs’ uptake [41]. Studies on GPs’ attitudes toward depression IMIs identified benefits for implementation, such as a facilitated diagnosis, reduced workload, individualized treatment, shorter waiting time, and support for more patients [42,43]. Furthermore, GPs reported barriers to implementing IMIs, such as infrastructure requirements, privacy concerns, low awareness of electronic mental health (e–mental health) interventions, low confidence in prescribing IMIs, lack of training, uncertainty about the evidence base [42], and concerns that computer treatments are too impersonal and would not meet patient needs [44].

### Objectives

The abovementioned insights refer to GPs’ perspective on IMIs and show that research on bPT is scarce. To the best of our knowledge, no study has yet investigated GPs’ perspectives on barriers to and facilitators for referrals to blended treatments. GPs may be more willing to refer to an IMI when it is combined with face-to-face psychotherapy, which is well known and trustworthy. At the same time, referrals might be facilitated by improved treatment availability and access to treatment options. However, given low GPs’ referral rates to psychotherapy and the novelty of blended treatments, an assessment of factors determining GPs’ referrals to bPT is needed. From this, implementation strategies can be designed to address these and maximize the uptake of bPT. This mixed methods study aimed to identify barriers and facilitators that influence the referral behavior of GPs to a bPT for MDD.

## Methods

### Study Setting and Design of the RCT

This study was part of a mixed methods German study arm of the European Project E-COMPARED, which was the first multicenter study that evaluated the clinical and cost-effectiveness of a blended depression treatment compared with treatment as usual across 8 countries. The objective of the German RCT was to evaluate the effectiveness of bPT based on cognitive behavioral therapy (CBT) for adults with a diagnosis of MDD compared with GP routine care. Further information on the RCT can be found in the study protocol [45].

The trial was approved by the Ethics Committee of the German Society of Psychology and registered in the German Clinical Trials Register (DRKS00006866). All GPs provided informed consent to participate.

Patients were recruited in GP offices in primary care and randomly assigned to the treatment conditions, bPT (n=86) and GP care (n=87). Professionals were not blinded. The bPT was conducted by psychologists in an outpatient university clinic. GP care consisted of (1) initial screening of MDD, informing about treatment options and referring depressed patients to the study; (2) conducting routine care for the control group; and (3) completing questionnaires about the diagnosis and treatment of patients at baseline and postassessment (13 weeks). All GPs received a print booklet with information about bPT and trial procedures as well as recruitment material (eg, flyers, displays, posters) to inform and motivate their patients. Their effort was reimbursed with EUR 100 (US $117.6) per partaking patient. Further motivating activities were a kickoff meeting, newsletter, phone calls, and onsite visits.

### Blended Internet-Based Psychotherapy

bPT was delivered as a short-term treatment (13 weeks) for MDD. It combined 6 internet-based CBT lessons (psychoeducation, behavioral activation, cognitive restructuring, problem-solving, physical exercise, and preventing relapse), daily assessments of mood, and cognitive and behavior-related parameters on a mobile app (eg, sleep habits, worries), as well as 6 biweekly face-to-face sessions with a psychologist and CBT therapist in training (Multimedia Appendix 1). Patients had free and password-encrypted access to a website with 10 weekly CBT-based web-based modules (text, videos, and exercises), mood graphs, a calendar, and a messaging system and independently edited the web-based modules at home. They received automated tailored web-based reminders. Therapists referred back and forward to web- and mobile-based content within face-to-face sessions to structure the treatment, monitored patients’ treatment course on the internet, provided weekly web-based feedback on exercises and progress, or wrote reminder and motivation messages. A detailed description and a case report are published elsewhere [46].

### Recruitment and Referrals

By the end of 2014, more than 1000 GPs were contacted through post, whose practices were located in the relevant catchment area for referrals to the university outpatient clinic (60 km). Overall, 137 GPs from 107 practices in Bavaria (Germany) agreed to refer to bPT. In the recruitment period from February 2015 to August 2016, 121 GPs referred to at least one patient, and 86 GPs had partaking patients in the RCT.

Successful referrals were operationalized as the number of patients who completed screening for MDD after being informed of it in GP offices. Owing to the study design, it was not possible to document the intended referrals onsite. Higher referral rates can be assumed; since some patients may not have followed the recommendation of their GP, the GPs had reported a high number of patients treated daily (mean 49, SD 22.84), and not all patients could be assigned to a GP.

The recruitment of GPs for this qualitative study took place shortly before the end of the study enrollment period (March to June 2016) by inviting 110 GPs from the trial population (those without study withdrawal) by email or telephone to participate in interviews. In total, 12 GPs (11%) took part in semistructured interviews. Of 110 GPs, 39 refused to participate; some were interested but had time constraints, and others could not be reached by telephone and emails with 3 reminders.

### Design of the Qualitative Study and Data Collection

We used a qualitative method with a theory-based approach to gain insights into the experiences and perspectives of GPs regarding their referral behavior to bPT. This exploratory method is a recommended approach to identify barriers and facilitators for implementing interventions [47].

The semistructured interview guide (Textbox 1) was based on the theoretical domains framework (TDF), which provides a comprehensive theoretical assessment of implementation problems and professional behavior [48]. The 14 domains (eg, knowledge and intention) represent potential determinants for the change of behavior of GPs during the implementation of bPT as a new referral option. It enables researchers to identify the hindering and facilitating factors to support the implementation of evidence-based interventions. Items were evaluated by 3 experts in clinical psychology, e–mental health, and qualitative research to enhance validity. When needed, guided prompts enabled gathering of information, and field notes complemented data collection. Pilot testing of the interview guide with the first interview (IT) did not require any adjustments.

**Theoretical Domains Framework and exemplary questions in the interview guide.**

**D01: Knowledge**

- What do you know about blended psychotherapy (bPT) and its effectiveness?

**D02: Skills**

- Which skills and competencies do you consider as necessary to refer patients to bPT?

**D03: Social/professional role and identity**

- Do you see referring suitable patients to bPT as part of your role? Is doing referrals to bPT incompatible or in conflict with professional standards or your identity?

**D04: Beliefs about capabilities**

- How difficult or easy is it to refer patients to bPT? How confident are you that you can overcome existing barriers?

**D05: Optimism**

- Based on your experience, how confident are you that referrals to bPT will run optimally?

**D06: Beliefs about consequences**

- What do you think are the benefits of bPT?

**D07: Reinforcement**

- Do you think the benefits of bPT for patients are sufficient to justify referral being part of your normal workflow?

**D08: Intentions**

- How much of a priority is bPT in the care of patients with depression? How essential is your personal requirement to refer patients?

**D09: Goals**

- How do you feel about the goal to implement bPT into the health care system in a way that you could refer to it in the future?

**D10: Memory, attention and decision processes**

- Is referring to bPT something you usually do or remember to do? What helps or would help you to remember?

**D11: Environmental context and resources**

- Do you think there are sufficient resources available if you start referring more patients to bPT? Which barriers outside your practice can prevent referrals?

**D12: Social influences**

- Did colleagues or patients/relatives ever prompt or encourage you to refer to bPT? Did they discourage your referral? What reactions do you expect?

**D13: Emotion**

- Do you think bPT is something patients are willing to participate in? Does thinking about referrals to bPT evoke any concerns?

**D14: Behavioral regulation**

- Are there procedures or ways of working that encourage referrals to bPT?

The sample size was determined by the low response rates to interviews at the end of the RCT enrollment period. Hence, the sample size and composition could not be planned. In total, 3 interviewers conducted 4 interviews with GPs. IT trained 2 interviewers with a Bachelor of Science in Psychology in using the interview guide and gave feedback to their first interviews. The interviews were conducted through telephone between May 2016 and June 2016, whereas the GPs were at the workplace (n=9), at home (n=2), or in the car (n=1). The average duration of the interviews was 56 min (SD 15.26; minimum=36 and maximum=78). Interviews were audio recorded and transcribed verbatim, based on a transcription guide. Data were pseudonymized using code numbers.

### Data Analyses

#### Qualitative Analysis

A qualitative content analysis was conducted, drawing on an inductive-deductive approach and using standardized methodical steps in qualitative research [49]. Codes were developed from the raw data and referred to themes relevant to the research question. The items were based on the TDF.

First, a list of codes was developed using 50% (n=6) of interview material, which involved identifying emerging themes, discussing the possible meanings of text excerpts, and arranging themes into categories within consensus meetings. Consensual coding was applied to enhance the quality of the coding process [50]; 2 independent coders developed and compared codes and discussed any differences to generate consensus on the codes.

Second, a further text retrieval was done to (1) label text related to the first draft of the code list, (2) specify subthemes, (3) add new emerging themes to the code list, and (4) target a higher level of abstraction of the codes. A paragraph was coded if it contained themes from one or more categories. IT gave written feedback to the coders regarding all labeled text frames to enrich the data interpretation through clinical expertise. The list of codes was revised and completed with additional definitions and exemplary statements. A preliminary code system, based on 50% (6 interviews) of the material, was developed and discussed in a consensus meeting among IT, SS, and the coders. They reached final agreement on code definitions and excerpts, the structure of the code system, and coding rules. This iterative research process aimed to finalize the code list to fit the data and to optimize the content and number of identified categories.

Third, all interview transcripts were independently coded by 2 coders in accordance with the code list. There was sufficiently moderate intercoder agreement with the coefficient kappa, k=0.71 [51]. Data saturation (a validity criterion of the codes list) was successfully reached, as each theme was mentioned by at least two GPs and no new themes emerged from the interview data. This indicated that no additional interviews should be conducted [52]. Key themes were defined as emerging themes mentioned by 100% of GPs.

Finally, to ensure the validity of the identified themes, the 12 GPs were questioned as to whether they agreed with the resultant barriers and facilitators via a survey. The themes were presented as a list. GPs agreed, on average, with 37 of 48 of all themes identified (mean 78%, SD 14%; minimum=44% and maximum=98%). The mean agreement rate per theme (ie, for each barrier or facilitator) was 78% (SD 19%; minimum=8% and maximum=100%). Thus, the identified barriers and facilitators yielded very good validation results.

#### Quantitative Analysis

A comparison of all barriers and facilitators and the percentage of all 12 GPs who mentioned these in the interviews and in the follow-up validation survey was conducted. Agreement rates per GP were analyzed.

Secondary quantitative analyses were conducted to evaluate whether there were any group differences in the identified barriers and facilitators. The interviewees were divided into 2 groups according to the following characteristics: “referral rates: high (n=4) versus low (n=8)” (cutoff by mean value=6), “experience years as licensed GP: high (n=6) versus low (n=6)” (cutoff by mean value=15), and “training in psychotherapy: yes (n=4) versus no (n=8).” A frequency table and descriptive statistics with 95% CIs were used to present differences in the barriers and facilitators between the abovementioned groups. Inferential statistics were not applied because of small samples and limited power. Power sensitivity analyses indicated that both equal (6/6) and unequal (8/4) small sample sizes would have the sensitivity to detect only a large proportional difference of 0.75 in a Fisher exact test and large effect sizes of *d*=1.96 (unequal) or 1.85 (equal) in a Mann-Whitney U test, each with a power of 80% and an alpha level of 5% (two tailed).

Independence tests of the interviewed sample (n=12) and the remaining RCT sample (n=64) were conducted using the Fisher exact test for categorical variables (because of cell frequencies <5) and using the *t* test or Mann-Whitney U test for continuous variables. The results of the nonparametric Mann-Whitney U test were reported because of unequal sample sizes, if both assumptions (normality of distribution and equality of variance) were not met. The test was applied for verification if the data were nonnormally distributed. All analyses were two sided, with an alpha level of 5%. Sensitivity power analysis for *t* tests, targeting unequal sample sizes (n=64 and n=12), an alpha level of 5% (two tailed), and 80% power, had yielded the sensitivity to detect an effect size of *d*=0.89.

Pearson correlations were conducted to determine the association between referrals and different continuous variables in the total RCT sample (n=76). The power analysis indicated that a sample size of 76 would have the sensitivity to detect an effect size of *r*=0.31, with a power of 80% (alpha level of 5%, two tailed) in a bivariate correlation.

The tool MAXQDA 12 (VERBI software, 2015) was used for the qualitative analysis and SPSS 25 (IBM SPSS Statistics, 2017) for the quantitative analysis. Power analyses were conducted using G*Power, version 3.1.9.7 [53]. A guideline for reporting qualitative studies (Consolidated Criteria for Reporting Qualitative Studies [COREQ] checklist) was applied (Multimedia Appendix 2) [54].

## Results

### Participant Characteristics and Referral Rates

Interviewed GPs were mainly men (8/12, 67%), with an average of 50.67 (SD 11.88) years and reported an average of 15.75 (SD 10.42) years of practice experience. Regarding their qualifications, 4 GPs had a license for psychotherapy and 9 had further education in psychosomatic basic care in addition to their university degree in medicine. GPs worked in both metropolitan (7/12, 58%) and rural (5/12, 42%) areas. Sociodemographic data for the remaining RCT sample (available for n=64) showed similar values, such as a proportion of 64% (41/64) being men, an average age of 52.14 (SD 8.73) years with 16.65 (SD 8.34) working years as a licensed GP. There were no statistically significant group differences in characteristics between the interviewed sample (n=12) and the RCT sample (n=64), all *P≥*.08 (see descriptive statistics in Table 1).

**Table 1:** Sample characteristics of participants.

Characteristics of GPs^a^		RCT^b^-GP sample (n=64), mean (SD)	Interviewed GP sample (n=12)			*P* value^c^	Low referrer interviewees (n=8)^d^, mean (SD)	High referrer interviewees (n=4)^d^, mean (SD)
			Mean (SD)	Minimum	Maximum			
**Age (years)**								
		52.14 (8.73)	50.67 (11.88)	29	75	.62	51.50 (13.70)	49.00 (8.60)
**Female, n (%)**								
		23 (36)	4 (33)	N/A^e^	N/A	>.99^f^	3 (38)	2 (50)
**Experience as licensed GP (years)**								
		16.65 (8.34)	15.75 (10.42)	1	31	.94	15.50 (10.58)	16.25 (11.67)
**Working in current practice (years)**								
		14.82 (9.47)	14.29 (9.36)	1	28	.86	13.19 (8.70)	16.50 (11.62)
**Training in psychosomatic care,** **n (%)**								
		51 (80)	9 (75)	N/A	N/A	.14^f^	5 (63)	4 (100)
**Training in psychotherapy,** **n (%)**								
		7 (11)	4 (33)	N/A	N/A	.09^f^	3 (38)	1 (25)
**Radius of patient catchment area (km)**								
		14.72 (11.09)	16.67 (12.26)	3	50	.59	11.25 (6.27)	27.50 (15.00)
**Number of daily treated patients**								
		48.80 (22.81)	52.00 (24.06)	20	100	.69	49.17 (18.55)	56.25 (33.51)
**Number of referrals to blended psychotherapy**								
		2.75 (3.06)	6.33 (9.42)	0	26	.66^g^	1.38 (9.2)	16.25 (11.27)
**Self-ratings on competences (range 1-5)^h^**								
	Diagnosing depression	3.81 (0.69)	4.08 (0.29)	4	5	.16^g^	4.13 (0.35)	4.00 (0.00)
	Depression treatment with psychotherapy	3.10 (0.93)	2.92 (0.90)	2	4	.54	3.00 (0.93)	2.75 (0.96)
	Supportive talk	3.76 (0.84)	4.08 (0.67)	3	5	.21	4.00 (0.54)	4.25 (0.96)
	Treatment with pharmacotherapy	3.33 (0.54)	3.00 (0.85)	1	4	.08	3.13 (0.64)	2.75 (1.26)
**Agreement with statements (range 1-5)^i^**								
	I am experienced in treating depression	3.75 (0.70)	3.67 (0.65)	3	5	.72	3.62 (0.74)	3.75 (0.50)
	I carry out depression treatment because of long waiting periods for specialist care	4.30 (0.98)	4.42 (0.67)	3	5	.70	4.38 (0.74)	4.50 (0.58)

^a^GP: general practitioner.

^b^RCT: randomized controlled trial.

^c^Statistics regarding group differences between randomized controlled trial (n=64) and interviewed sample (n=12). A *t* test was used, if not otherwise specified. The results of *t* tests were verified if one requirement was not fulfilled.

^d^Groups were split by mean value (low referrers mean <6; high referrers mean ≥6).

^e^N/A: not applicable.

^f^The Fisher exact test was used for categorial variables.

^g^The Mann-Whitney U test was used if both assumptions were not met because sample sizes are unequal.

^h^Scale from quite low (1) to quite strong (5).

^i^Scale from not applicable (1) to very applicable (5).

The patients treated with bPT (n=86) were aged on average 43.22 years (SD 13.07; range 19-70), mostly women (61%), highly educated (56%), and employed (74%). Overall, 71% had no prior experience with psychotherapy.

Of 698 successful referrals to bPT, 321 could be assigned to 121 GPs in the RCT. The majority of RCT-GPs (92/121, 76%) referred fewer than 3 patients to bPT during the 18-month study period, indicating a ceiling effect. However, there was a large variation between GPs in the number of referrals made (RCT sample: mean 2.65, SD 3.92; minimum=1, maximum=26; interview sample: mean 6.34, SD 9.42; minimum=0, maximum=26), and interviewed GPs had higher referral rates than those in the RCT. Table 2 shows the frequency distribution of GPs’ referrals to bPT in the interviewed sample (n=12) and the RCT sample (n=121).

**Table 2:** Frequency distribution of GPs’ referral rates to blended psychotherapy.

Referral rates		Randomized controlled trial-GP^a^ sample (n=121), n (%)	Interviewed GP sample (n=12), n (%)
**Number of referrals per GP**			
	0	0 (0)	1 (8)
	1	63 (52.1)	4 (33)
	2	29 (24)	2 (17)
	3-5	20 (16.5)	1 (8)
	6-8	5 (4.1)	2 (17)
	12	1 (0.8)	0 (0)
	21	1 (0.8)	0 (0)
	26	2 (1.7)	2 (17)

^a^GP: general practitioner.

A significant moderately negative correlation was found between the number of referrals made and self-rated competence in delivering pharmacotherapy (*r*(73)=−0.31; *P*<.001), that is, GPs with higher self-confidence in delivering pharmacotherapy were less likely to make referrals to bPT. Correlations between referrals and demographics, medical experience, competence ratings, and depression management statement agreements were otherwise nonsignificant (Multimedia Appendix 3).

### Qualitative Findings

Altogether, 19 barriers and 29 facilitators were identified and categorized into 4 main areas: (1) the *general practitioner*, (2) the *patient*, (3) factors influencing the routine in the *GP practice*, and (4) factors relating to *sociopolitical circumstances* that influence the implementation of a bPT for MDD in Germany as well as the referral process. In total, 77% (37/48) of the identified themes were mentioned by at least five or more interviewed GPs (42%), whereas 44% (21/48) of the themes were mentioned by at least eight GPs (67%).

#### Barriers to Referrals

Most emerging barriers (11/19, 58%) were assigned to the patient level. Five barriers (5/19, 26%) were named by at least nine GPs (75% of interviewees), whereas 2 barriers (2/19, 11%) were mentioned by all 12 GPs. These latter 2 key barriers are presented in the following section with a quotation illustrating the physicians’ experiences, whereas all 19 barriers are described with a definition and supporting quotations in Table 3.

**Table 3:** General practitioners’ perceived barriers for referrals to blended internet-based psychotherapy (bPT) for depression.

Barriers (n=19)		GPs^a^ (n=12)		Definition	Supporting quotations
		n (%)^b^	κ^c^		
**Level of *general practitioner* (barriers n=4)**					
	Little knowledge about internet-based interventions	12 (100)	22	The GPs know little about the content and procedures of internet-based interventions or bPT^d^. Furthermore, they declare to have no profound scientific knowledge about their clinical effectiveness	“To date, I don’t know anything about the efficacy of internet-based interventions.” [GP059]
	Lack of feedback on referral or treatment response	9 (75)	13	The GPs need more feedback on the effectiveness of bPT for a patient, and if necessary, recommendations for further treatment. Feedback is claimed to be an essential component of the communication between GPs and other professionals in health care	“It’s important to be immediately informed of its effects on the patient [...] whether bPT was successful. Or conversely, what measures should be taken if the patient became seriously ill.” [GP084]
	Skepticism toward the internet-based intervention	6 (50)	10	The physicians are skeptical of the quality of internet-based interventions for depression. As a result, they prefer other treatment options for patients	“I would prefer traditional psychotherapy if there could be shorter waiting periods, because it’s more sustainable, a long-term treatment and more personal.” [GP026]
	Lack of habit and routine	5 (42)	10	Referrals to bPT are not a habit and not yet normal for the everyday working routine. The integration of a new procedure takes time and is a complex process. The reasons for this include lack of time, lack of familiarity with the program, or no coverage by medical insurance	“The routine is still developing. It’s not part of my role yet. [...] Referrals to bPT have not yet been embedded in my routine and my regular work, so to speak.” [GP043]
**Level of *patient* (barriers n=11)**					
	Lack of familiarity with technology, internet, and media	12 (100)	22	The GPs assume that some patients are not confident in using computers, mobile phones, and new technology. They might not be familiar with these devices or have little experience using them	“I imagine not all patients are tech-savvy. Even when you provide them with a smartphone, I think, they’re scared of not being able to use new technologies.” [GP007]
	Disease-specific contraindication	10 (83)	20	The GPs assume that patients with certain diagnoses are not suitable for the internet-based intervention, for example, severe forms of depression, chronic clinical course, suicidality, lack of introspection skills, personality disorder, high comorbidity, or psychotic symptoms	“A referral is not an option if there is an acute suicide risk, a borderline disorder or eating disorders.” [GP059]
	Reservations and skepticism toward technology in treatment	9 (75)	14	The patients are skeptical of the treatment and whether therapy can be effectively implemented and delivered through internet technology	“Patients say ‘Internet? That works for depression treatment? Really?’ They are quite skeptical.” [GP097]
	Lower suitability for physically limited or older patients	8 (67)	9	Internet-based interventions for depression are often considered less suitable for patients with physical limitations. GPs would not refer older patients to internet-based interventions	“It’s clear that older patients, who have bad eyesight, won’t sit in front of their screen and do an internet-based intervention.” [GP059]
	Reservations regarding data safety	7 (58)	12	Patients express reservations regarding data safety with internet-based interventions for depression or this is assumed by the GP	“Patients are often very anxious about data safety when offering something via the Internet. They don’t believe me when I tell them that their information stays confidential.” [GP084]
	Lack of internet access or a computer	5 (42)	7	Internet-based treatment is refused or cannot take place because patients do not have the technical equipment (mobile phone and computer with internet access)	“Well, apart from not having access to the internet and a lack of trust in data security, I can’t think of any big barriers.” [GP012]
	Limited therapeutic relationship or personal contact	5 (42)	9	Internet-based intervention does not provide enough therapeutic contact for the patient. Interviewees mention that a good therapeutic relationship and good rapport cannot arise through internet-based interventions	“Maybe for some the internet-based intervention is too impersonal. Some patients might still prefer more human contact and a closer relationship.” [GP007]
	Fear of stigmatization	3 (25)	4	Having depression and starting bPT causes unpleasant feelings within patients. They fear the consequences of a psychological disease in society	“When you name the disorder, it is often hard for patients to accept it. When you say ‘Listen, the depression has to be treated and there is bPT,’ the patient often doesn’t like it.” [GP059]
	Personal effort required	3 (25)	5	Contributing actively and independently to the treatment can be burdensome for patients. It can be especially challenging for those with depression	“I say to the patients: ‘You have to work, you use your PC at home.’ [...] It involves personal effort and it is not as comfortable as sitting in the GPs’ office and going home afterwards.” [GP046]
	Language barrier	3 (25)	4	Patients who are not able to speak German, which is needed for the intervention, cannot take part in the internet-based intervention. The GPs believe there is a need for translations into other languages for immigrants and refugees	“One disadvantage is that the intervention excludes refugees and immigrants because of the language. They’re a big patient group. They need a translator.” [GP026]
	Little room for individualized treatment or personal issues	2 (17)	4	The limited number of face-to-face sessions and the therapists’ orientation toward web-based modules leave little therapeutic room for personal issues and individualized treatment	“I had a patient that struggled with bPT. The face-to-face sessions were too attached to the internet part and he wanted to talk more about himself. It was too rigid for him.” [GP007]
**Level of *GP practice* (barriers n=1)**					
	Lack of time for explanations about the availability of bPT	3 (25)	3	The GPs and their office staff lack temporal resources to explain the new bPT that is available to patients	“When the counselling for depression treatment takes too much time, when I spend too much time explaining […] that there’s something new and how it works, then I won’t have enough time.” [GP043]
**Level of *sociopolitical circumstances* (barriers n=3)**					
	Low awareness of bPT as a therapeutic method	7 (58)	11	Public knowledge and awareness of internet-based interventions and bPT for depression is limited, and this reduces the willingness to use bPT	“Nobody asked me or reinforced to me that I should refer to bPT. That’s too early. It isn’t well-known enough yet.” [GP046]
	No reimbursement by health insurance	6 (50)	9	Health insurance companies do not cover the reimbursement of internet-based interventions	“Health insurers should pay for it and there should be contingencies” [GP007]
	Organizational, bureaucratic, and legal requirements for care providers	5 (42)	6	Organizational, bureaucratic, or legal obstacles can avert the referral to bPT for depression. This includes the interference of health insurance companies, shortage of money, legal requirements, etc	“The bureaucracy and the legal requirements of §12 SGB V, that asks for ‘efficiency principles’, as well as the entire organization of the Association of Statutory Health Insurance Physicians are aggravating factors.” [GP046]

^a^GP: general practitioner.

^b^The percentages give the proportion of all 12 general practitioners who mentioned the barrier.

^c^Number of excerpts (κ) show the number of statements regarding a barrier on a code level.

^d^bPT: blended psychotherapy.

All interviewed GPs reported feeling poorly informed about the content and the procedure of bPT and having little knowledge about the scientific evidence of the treatment, as one GP outlined:

I know nothing about the effectiveness of bPT. I have not read anything about it in GP journals.

GP098

All respondents agreed that patients who are not familiar with computers or smartphones and have little experience with technology might struggle with the technology-based treatment:

There are still people today who are not familiar with computers, especially in rural areas.

GP053

#### Facilitators for Referrals

The analysis revealed more facilitating than hindering factors on the levels of the GP as a person, GP practice, and sociopolitical circumstances. Of the identified 29 facilitators, 16 (55%) were mentioned by at least 75% (n=8) of GPs and 2 (7%) by all interviewees. Below, these 2 key facilitators are presented with an outlining statement. All 29 facilitators are described with a definition and supporting quotations in Table 4.

**Table 4:** General practitioners’ perceived facilitators for referrals to blended internet-based psychotherapy for depression.

Facilitators (n=29)		GPs^a^ (n=12)		Definition	Supporting quotations
		n (%)^b^	κ^c^		
**Level of *general practitioner* (facilitators n=11)**					
	No conflict with GP’s role	12 (100)	16	Referral to a bPT^d^ is not in conflict with professional standards and the GP’s role identity. Interviewees integrate the referral into their job role without problems	“I don’t see a role conflict. As a GP you’re not a specialist for everything, therefore it belongs to my daily routine to refer patients to a specialist. My competence is recognizing patients being in need of a specialist.” [GP084]
	High level of self-efficacy regarding the referral	11 (92)	15	The GPs feel able to do the referral to bPT in a promising and successful way. They describe no difficulties for the referral process	“The referral to bPT is not very complicated. It already works quite well.” [GP084]
	Optimistic attitude	11 (92)	15	The interviewees are open toward the new internet-based approach. Physicians are confident that the bPT will work well and the treatment will be successful	“I am very confident that bPT can be a great support. Definitely.” [GP007]
	Support through information and training	11 (92)	19	The GPs would benefit from more information, further education and training, and the opportunity to try out the tool themselves. This increases the likelihood of a referral	“I think a 2-3 hours training session would be nice. A short introduction into the disorder, the diagnostic process of the GP and information about the blended treatment.” [GP026]
	Positive self-appraisal regarding own skills	9 (75)	10	The GPs rate their own skills as very high which are needed for a successful referral. Those include, for example, the diagnosis of depression and counseling techniques	“The main skill is actually interviewing. I learnt most the things by doing further educational courses. As a GP, my main weapon is talking.” [GP084]
	Positive beliefs about treatment success	9 (75)	12	The physicians expect the blended depression treatment to be associated with positive consequences for the patient	“I can imagine, that a certain group of patients could benefit a lot from it. [...] I think it is a step forward.” [GP084]
	Positive attention and decision-making process	9 (75)	16	Positive attention and decision-making processes are supported by the following: the referral option is remembered, GPs get reminders (flyers and mails) from the therapy institution, and patients give positive responses to the treatment	“I really remember it all the time. I have the flyer on my desk and every patient that seems suitable to me gets one from me.” [GP026]
	Expectation of social reinforcement	9 (75)	9	The GPs expect positive reactions within their social environment for the referral of patients to bPT	“I would expect interest and curiosity about bPT from others.” [GP012]
	Perception of patients’ consent	9 (75)	9	The physicians feel that patients are willing to take part in blended depression treatment. They expect patients to give consent when they express a referral offer	“Yes, patients are willing to participate in the bPT.” [GP007]
	Positive emotions	8 (67)	8	Pleasant emotions arise when the GPs consider referring a patient to bPT for depression	“It feels good to be able to refer a patient to bPT.” [GP007]
	Personal contact with the therapists and provider	7 (58)	11	Personal contact with therapists and staff of the provider reinforces the GP’s referral behavior	“It was helpful to know the staff.” [H026]
**Level of *patient* (facilitators n=9)**					
	Perceived patient suitability (eg, well-educated or young)	12 (100)	22	bPT is an up-to-date intervention, which is close to patients’ everyday media use. It is especially suitable for people of a younger age and a medium to high level of education	“Patients with a higher educational level are more suitable.” [GP012]
	Intervention for minor-to-moderate depression or dysthymia	10 (83)	22	The GPs perceive bPT to be appropriate for patients who are affected by minor-to-moderate depression or dysthymia	“I would refer patients suffering from mild to moderate depressive disorders. I don’t consider bPT to be sufficient for severe forms.” [GP012]
	Motivation and willingness for treatment	7 (58)	13	The interviewees are ready to recommend the blended treatment if the patient shows motivation and willingness to start the treatment	“If a patient shows interest and wants to be informed about the bPT, I am open and ready to recommend the offer.” [GP012]
	Smaller inhibition threshold and barrier for usage	7 (58)	15	Patients have fewer inhibitions about taking part in internet-based interventions for depression and taking advantage of this offer. One can reach new patient groups, who were not previously open for treatment	“Another facilitating factor is maybe the low threshold, particularly for younger patients, to contact a psychologist online.” [GP012]
	Time- and location-independent internet-based intervention	6 (50)	8	The internet-based components of the treatment are neither bound to place nor to time, so they can be easily integrated into daily life. The patient works through the internet-based modules in a flexible way	“The advantage is surely that bPT is not time bound, at least for the internet-based part of the treatment.” [GP046]
	Anonymity	5 (42)	7	Patients also participate because of the given anonymity of the web-based section of bPT and appreciate this anonymity	“The anonymity is an advantage. If patients can register on their own on an online platform, it will surely help. Some just have this threshold and are frightened to speak about their problem in face-to-face contact.” [GP007]
	Technical affinity	4 (33)	4	Working with technical tools and new media is an incentive to join internet-based interventions for depression. People who have an affinity for technical tools are attracted to this new technology	“Younger patients, who are often technophiles, are especially interested in bPT, I think.” [GP012]
	Preventive approach for subclinical symptoms	3 (25)	7	The GPs believe the bPT is suitable for subclinical symptoms of depression. It can also be used for prevention	“I think it is an in-between treatment. I need it for people diagnosed with a depressive adaptation disorder who are still at a beginning point with subclinical symptoms.” [GP007]
	Short duration of treatment	2 (17)	3	The treatment only lasts 13 weeks, which is a manageable amount of time for patients	“The behavior-centered working, six sessions in a short-term, therefore a manageable thing. I like that.” [GP084]
**Level of *GP practice* (facilitators n=3)**					
	Methods facilitating GPs’ work and referral process	11 (92)	40	Working routines that help GPs to treat depressive patients and to refer them, for example, information flyer, poster in the waiting room, newsletter, standardized referral documents, digital feedback about diagnostic and treatment findings	“Concerning one patient, I underestimated the severity of the disorder and I received diagnostic feedback from his therapist. I called a neurologist for an appointment in the near term. I saw this positively since I don’t claim that I can recognize everything.” [GP084]
	GPs’ perception of a high demand	11 (92)	14	Additional treatment options for the referral of depressive patients are strongly needed in GPs’ offices	“There is an increased prevalence of depression, especially in younger patients. Those providing outpatient therapy are overloaded. There is a high need.” [GP098]
	Saving GPs’ office resources	7 (58)	14	Referrals to bPT saves GPs’ resources. Patients can apply for therapy on their own via an web-based link. Physicians do not have to bridge the time with a treatment in their own office until a patient starts a traditional treatment	“Referrals to bPT are relatively time saving, don’t need many resources, are quickly executed and actually quite feasible.” [GP012]
**Level of *sociopolitical circumstances* (facilitators n=6)**					
	Short waiting time for internet-based intervention	11 (92)	38	The waiting time for internet-based interventions is essentially shorter than for traditional face-to-face psychotherapy. Depressive patients can be treated promptly, and as such, psychological strain is reduced	“The main advantage is that there’s no time delay. You can offer your patient a treatment immediately.” [GP012]
	Quick and easy availability	10 (83)	18	Internet-based intervention is quickly and easily accessible for patients without any large organizational and temporal effort	“Everybody can start the treatment very easily at home and almost immediately. The patient doesn’t need to arrange an appointment for the first contact, he can start online.” [GP059]
	Additional therapy approach as new pillar in health care	9 (75)	21	The GPs benefit from the provision of an additional up-to-date treatment offer in the health care system. bPT can close the care gap and has the potential for nationwide coverage	“bPT closes a gap in our range of therapeutic treatments. It is a very good additional option. Access to psychotherapy is limited and everything additional is something positive.” [GP026]
	Integration within guidelines as evidence-based treatment	6 (50)	11	bPT for depression should be established in the health care system as an intervention with approved evidence. The GPs should be informed about the guidelines and the recommendation of bPT as a treatment for depression	“Well, GPs are treating patients according to guidelines and it would help, if bPT were to be integrated into these guidelines. We have to be sure, that we can work with this treatment without hesitation.” [GP053]
	Media coverage improves awareness and evaluation of bPT	3 (25)	5	Media reports about the possibility of bPT for depression as an approved therapeutic approach improves GPs’ and patients’ perceptions of it and their willingness to use it	“A kind of public relation would be helpful. I benefit from the media coverage. If my patients get aware of this bPT through TV reporting, then I can say ‘Yes, I’m a part of it, too’.” [GP084]
	Bridging the waiting time for traditional psychotherapy	2 (17)	4	Long waiting times for specialized therapeutic care are a burden for the patient. Short-term bPT for depression can be used while patients wait for the start of a long-term traditional therapy. It helps to prevent the deterioration of symptoms	“I wonder if I should refer my patient first to your bPT short-term treatment and hope it will help, or should I choose a referral to a long-term psychotherapy with long waiting lists. I have to admit, I sometimes did both.” [GP026]

^a^GP: general practitioner.

^b^The percentages give the proportion of all general practitioners who mentioned the facilitator.

^c^Number of excerpts (k) show the number of statements regarding a facilitator on a code level.

^d^bPT: blended psychotherapy.

All interviewed GPs perceived no role conflict between the referral to bPT and their professional identity and standards:

I see the referral to bPT as part of my role. I see it as a welcome opportunity to be able to offer the patient a quick and uncomplicated means of assistance here in the practice.

GP059

Each GP identified patient groups for whom the bPT suits well. They characterized these patients as those who are young and highly educated, used to work with computers and smartphones, and ready to integrate them into their treatment:

I think it is definitely suitable for younger patients and also for patients who are already at work and do not have that much time.

GP083

### Quantitative Findings

#### Follow-Up Validation of Qualitative Findings

The follow-up assessment of the identified themes within the qualitative interviews showed a very high agreement rate between all 12 GPs of at least 75% for 34 (71%) of the identified barriers and facilitators. In total, 44 of 48 (92%) themes yielded an agreement rate of at least 58%, and 3 (6%) themes were agreed by all GPs. Only 2 (4%) themes had an agreement rate less than or equal to 25% of GPs.

*Low awareness of bPT as a therapeutic method* was the barrier, and *integration within guidelines as evidence-based treatment* and *short waiting time for internet-based intervention* were the facilitators, which resulted in the highest agreement rates. *Patients’ fear of stigmatization* was the barrier, and *GPs’ expectation of social reinforcement* was the facilitator with the lowest agreement rates.

**Figure 1:**
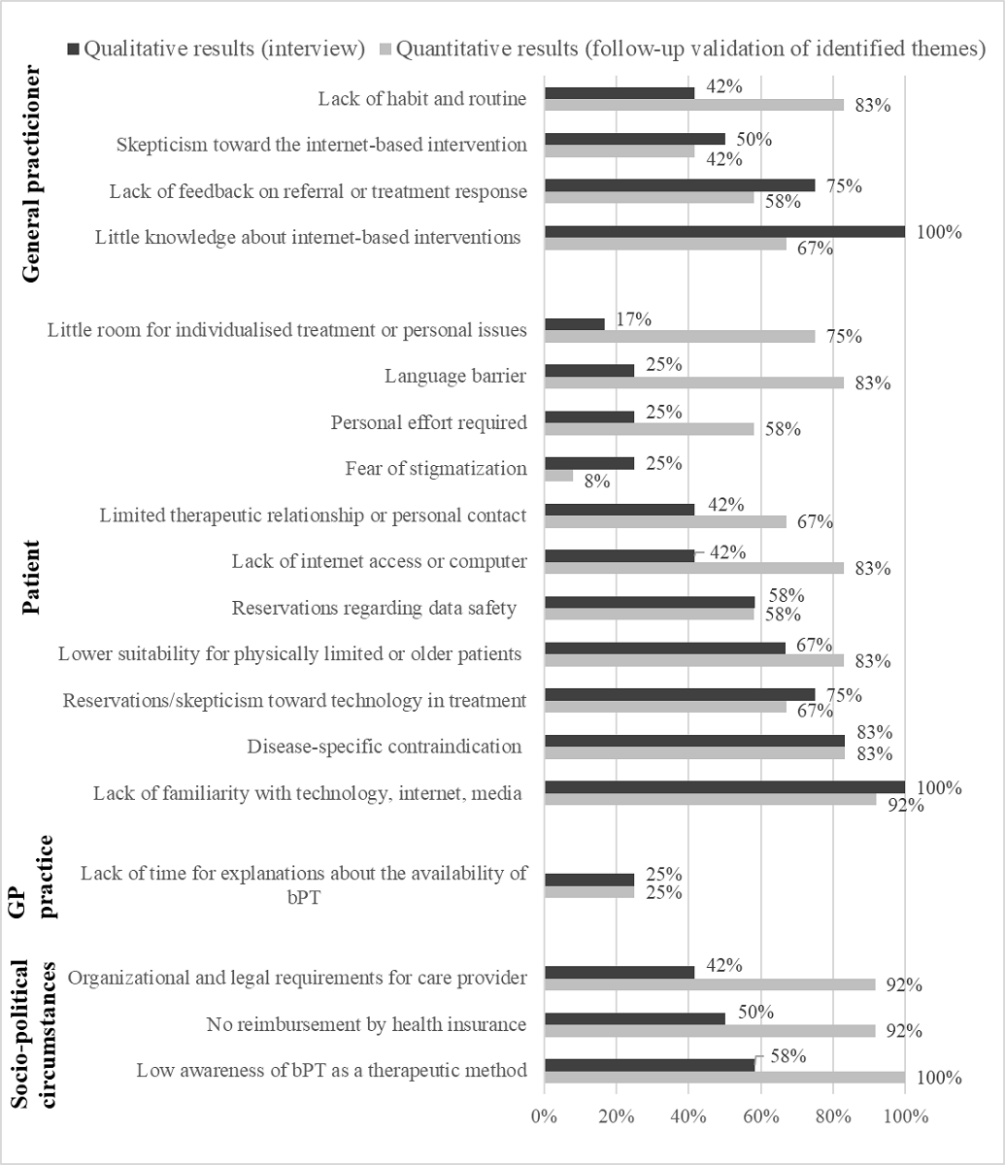
Frequency graphs of barriers for referrals to blended psychotherapy (bPT) mentioned in the interviews and in the follow-up assessment. GP: general practitioner.

All barriers, and the percentage of all 12 GPs who mentioned these in the interviews and in the follow-up validation survey of qualitative findings, are listed in Figure 1. The average proportion of GPs who mentioned these 19 barriers in the interview was 53% (SD 25%; minimum=17% and maximum=100%), whereas the mean agreement rate for barriers in the quantitative follow-up survey was 69% (SD 24%; minimum=8% and maximum=100%). When these 19 identified barriers were presented to GPs as a list, 53% obtained higher agreement rates than in the interviews.

**Figure 2:**
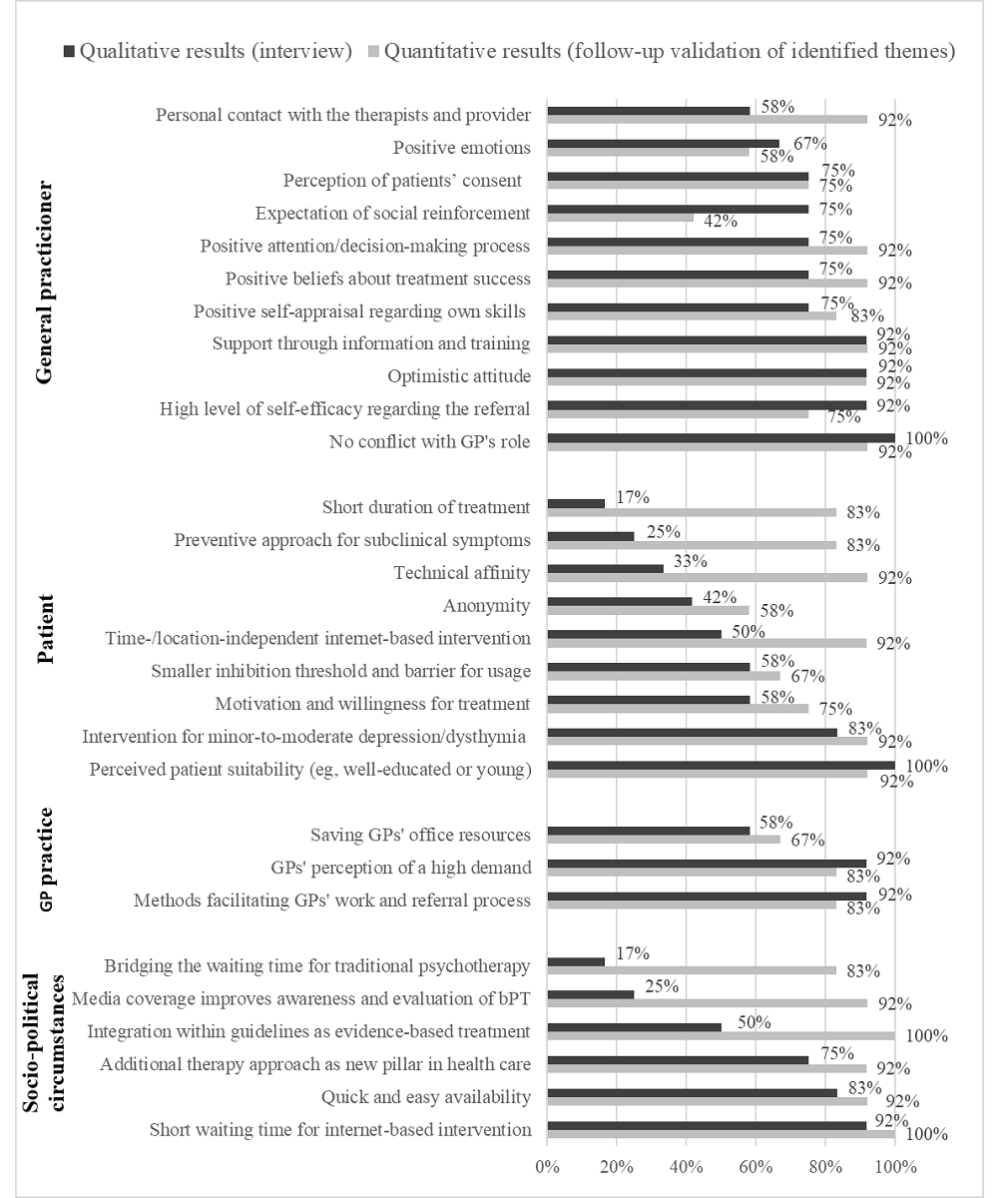
Frequency graphs of facilitators for referrals to blended psychotherapy (bPT) mentioned in the interviews and in the follow-up assessment. GP: general practitioner.

All enabling factors and the percentage of all 12 GPs who mentioned the facilitator in the interview and the follow-up validation survey of qualitative findings are listed in Figure 2. The average proportion of GPs who mentioned these facilitators in the interview was 66% (SD 25%; minimum=17% and maximum=100%), whereas the mean agreement rate for facilitators in the quantitative follow-up survey was 83% (SD 14%; minimum=42% and maximum=100%). Overall, 66% of these 29 facilitators received higher agreement rates from GPs when presented as a list.

#### Differences in the Barriers and Facilitators Perceived by GPs With Different Characteristics

Frequency tables indicated there were differences between GPs with different characteristics (low or high referrers, low or high GP experience, and no or yes psychotherapist) in terms of whether they mentioned particular barriers and facilitators. Descriptive statistics and 95% CIs can be found in Multimedia Appendix 4.

**Figure 3:**
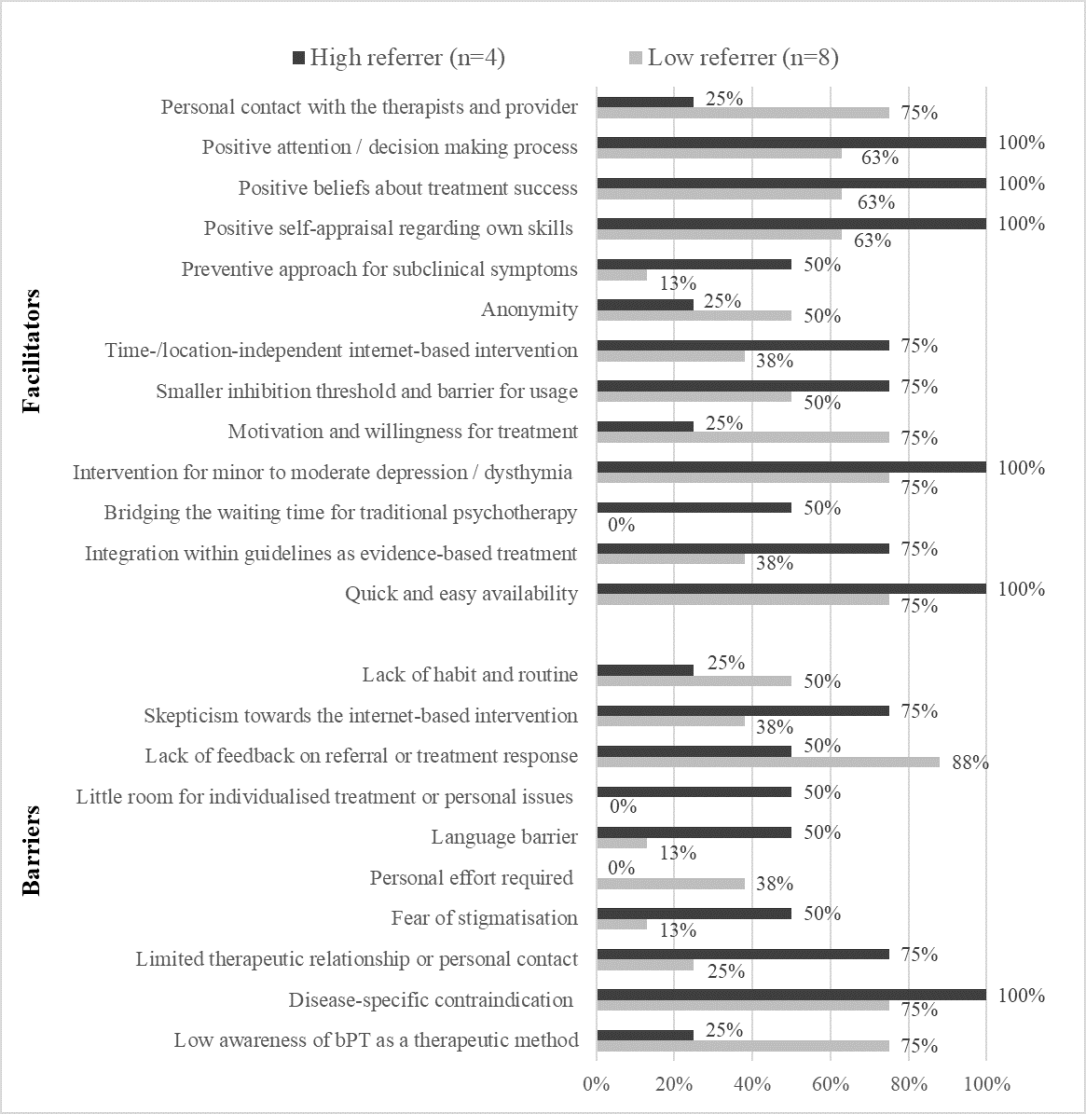
Barriers and facilitators mentioned by high versus low referrers with group differences of ≥ 25%.

Barriers (10/19) and facilitators (13/29) with a discrepancy of at least 25% among GPs with high versus low referral rates are shown in Figure 3. Those mentioned by more high referrers, with a difference of 50%, included *bridging the waiting time for traditional psychotherapy*, *little room for individualized treatment or personal issues*, and *limited therapeutic relationship or personal contact*. Low referrers more frequently mentioned with a difference of 50% *low awareness of bPT as a therapeutic method*, *personal contact with the therapists and provider*, and *patients’ motivation and willingness for treatment*. Such meaningful differences were not found when comparing GPs with high or low experience and training or no training in psychotherapy.

The GPs with low referral rates mentioned fewer barriers (mean 9.75, SD 1.83) and facilitators (mean 18.25, SD 4.13) than those with high referral rates (B: mean 10.50, SD 2.38; F: mean 21.00, SD 3.92). Both GP groups mentioned, on average, more facilitators than barriers.

## Discussion

### Principal Findings and Comparison With Prior Work

This mixed methods study investigated facilitators and barriers for referrals to bPT for depression from the perspective of GPs. The results should be interpreted in light of the fact that the sample was limited, small, self-selected, and unlikely to represent the full range of all GPs’ experiences (because of low response rates). Although referral rates were quite low, they differed between GPs. The lower the RCT-GPs rated their pharmacotherapeutic skills, the more referrals they made. Interviewees referred more than double the number of patients than the RCT-GPs.

All 29 facilitating and 19 hindering factors were identified on the levels of GP, patient, GP practice, and sociopolitical circumstances. The most frequently named barriers by the interviewed GPs concerned the use of new technologies within blended treatments, as most assumed that some patients would not be familiar with internet and technology or would be skeptical and uncertain as to whether an internet-based intervention could help them. These findings are in line with earlier research on barriers to the use of bPT by psychotherapists [34,35,55,56] as well as IMIs by clinicians (psychiatrists or GPs or psychologists) [57] or by GPs [42]. Furthermore, GPs’ concerns regarding data safety were also mentioned in previous research on bPT by GPs [58] and psychotherapists [34,35,55] as well as on IMIs by GPs [42] and psychotherapists [59].

The interviewed GPs reported not to be familiar with bPT, to have little knowledge of it, and to prefer more information and training. This is in accordance with the results of studies on bPT in GPs [58] and psychotherapists [56] and stand-alone IMIs in GPs [42,60] and health professionals [61]. GPs also asked for more feedback and professional exchange regarding the treatment, consistent with requests by professionals in previous research on IMIs [57] and with GPs’ reported facilitators for referrals to IMIs [41].

GPs had positive beliefs about the treatment success of bPT as well as high self-efficacy levels for patient referrals, contrasting previous findings on IMIs for depression in GPs [42]. This might be because of a higher level of trust in familiar face-to-face treatments or to the scientific setting of the conducted bPT, which reduced uncertainties. The finding that GPs feel no conflict with their role was in contrast with psychotherapists’ perceptions [36] but could potentially be explained through their gatekeeper function [40]. Other research suggested that GPs valued the feeling of being more skillful and professional when they blended IMIs with their depression treatment [58].

Further facilitators for referrals mentioned by the interviewed GPs relate to the health care system (eg, shorter waiting time, simple access, closing the treatment gap, feeling able to react to the high number of depressive patients in a better way) and are in line with research on IMIs with GPs [41,42] and bPT with GPs [58] and psychotherapists [34,35]. At the same time, GPs reported barriers such as little familiarity with treatment, missing reimbursement, and legal requirements, which correspond with research of IMIs in GPs [42] and of bPT in psychotherapists [34,55].

GPs’ attitudes that patients are suitable for bPT if they are familiar with modern technologies or are affected by minor-to-moderate depression were in line with previous research on bPT with psychotherapists [34,35,56] and GPs [58] and on IMIs with professionals [57,61]. The latter corresponds with treatment guidelines for depression [7,8]. Nevertheless, there is evidence that patients with severe forms can benefit from IMIs compared with untreated controls [62]. Furthermore, GPs judged suicidality and psychotic symptoms as a contraindication for bPT, corresponding with the attitudes of psychotherapists [34,56], despite studies showing that suicidal thoughts [63,64] and positive psychotic symptoms [65] can be targeted successfully with IMIs.

The finding that GPs perceived younger patients to be more suitable for bPT compared with older patients is in accordance with research on IMIs in clinicians [57] and GPs [58] and on bPT in psychotherapists [34,35,55,56]. However, a meta-analysis indicated that older patients significantly profited from a stand-alone IMI for depression and to an even greater extent compared with younger patients [62].

The findings can be interpreted in the context of prior systematic reviews on barriers and facilitators and help to develop implementation strategies. First, they align with the 7 stages of the implementation process of an intervention (eg, physician, patient, and system barriers), which were identified using 256 publications to categorize barriers to optimal clinical practice in health care [66]. These stages could support the implementation of referrals to bPT in practice. Second, the findings fall under important groups of determinants relating to the use of e–mental health interventions [67] and suggest that these are considered valid by GPs in relation to bPT. The authors recommend that implementation practitioners consider such determinants to achieve better implementation results and use these to design and apply specific implementation activities. The research project ImpleMentAll aims to find evidence for such an intervention (the ItFits-toolkit) through the development, application, and evaluation of tailored implementation strategies in ongoing eHealth implementation initiatives [68].

### Limitations and Strengths

This study has noteworthy limitations, such as low response rates of GPs to interview invitations (RCT-GPs: 12/110, 11%) and a limited sample size. This meant that further sampling to check the consistency of findings was not possible, and having a small sample size may have produced fewer themes and nuances in GPs’ views than the 20 to 30 interviews suggested by guidelines [69]. Yet, sufficient data saturation was reached with 12 interviews. All themes were mentioned by at least two GPs and 45 themes (94%) were mentioned by at least three GPs, which suggests that the findings covered important topics. This is in line with a study that reached saturation with the 12th interview of 60 interviews [70]. Nevertheless, the results of this study represent the perceptions of a small number of interviewees and, as such, may not be representative or generalizable for the whole RCT or primary care GPs. For example, interviewed GPs conducted twice as many referrals (and with 3 times the amount of variance) as the larger sample of RCT-GPs, indicating a self-selection bias of the interview participants toward the Central Research Question. Interviewees may have had a higher motivation and openness to referrals and possibly perceived different barriers and facilitators than the group of RCT-GPs. Concordantly, such differences were found within the interviewed GPs between those with high and low referral rates. However, the mean value of the interviewees was biased by 2 participants having the highest referral rates (each 26), and the average referrals of the interviewees as well as RCT-GPs were both rather low (difference in means was 3.69). The low referral rates resulted in a ceiling effect and limited the mean difference between the groups with low and high referrals. This meant that GP group members with high referral rates potentially experienced referrals to bPT similarly to the low referral group, negatively impacting the ability to make meaningful between-group comparisons. The interpretation of quantitative group comparisons should be considered with care because of the small and unequal distributed subsamples. To avoid this limitation, future studies should plan the composition and size of the sample a priori. Furthermore, as low referral rates indicate a low degree of practice and the referral behavior occurred within a standardized RCT setting with an unfamiliar intervention, the perceived barriers and facilitators may not be representative of routine practice. As the GPs participated in the RCT, they may have been biased toward more positive views regarding technology-based treatments and research, indicating a selection bias. GPs in routine care and/or with more experience with bPT might express other attitudes and views. On the other hand, when implementing bPT as a referral option in primary care, these early insights will be important for the outcome. Further limitations include the failure to register the intended referrals by GPs. Referral rates per GP relied on patient self-report screening data and may reflect the successful number of referrals rather than the actual intended referrals.

The strengths of this work included the mixed methods approach, which enabled the research team to generate in-depth findings by using a theory-based interview guide, and validated qualitative findings with a survey. The consensus and iterative approach is used to develop codes, and independent coding with a moderate interrater agreement enhanced rigor in producing the results. Identifying GP subgroups and comparing differences between these provided an indication of whether barriers or facilitators differed in their importance to different GPs.

### Implications for Clinical Practice and Future Research

Findings relating to barriers and facilitators could be used to design implementation strategies to support the integration of bPT as a referral option in clinical practice. The TDF is associated with the Behavior Change Wheel framework, which connects theoretical domains to 3 broad drivers of behavior: capability, opportunity, and motivation (COM-B model). It also connects these to specific intervention options [48,71], describes step-by-step how implementers can develop measures for behavioral change, and supports theory-based decision making [71]. Therefore, the study findings can be used to derive helpful, theory-based practical tips for developing effective intervention strategies for the implementation of bPT. Possible interventions include training and communication activities that can address the need for knowledge, attitudes, and misconceptions about bPT and expected patient reactions; legally required sophisticated IT solutions that reduce uncertainties regarding technology use and data safety; and stakeholder and policyholder involvement to drive the necessary adaptation of reimbursement or treatment guidelines.

In addition, future studies might explore barriers and facilitators in larger samples, invite GPs that have referred to bPT in routine care, and consider different delivery options of digital interventions (eg, guided IMIs). Furthermore, the roles of GPs as referrers to or users of blended treatments for different mental health disorders should be evaluated.

### Conclusions

This study provides insights into barriers and facilitators determining GPs’ referral behavior in relation to bPT for depression. The results indicate that GPs perceive bPT as an additional and valuable treatment delivery format. Having a central position in depression treatment, they experience positive consequences for their own professional group and for patient care when they are able to use bPT as a referral option. Thus, GPs appear to be ready as stakeholders to integrate digital interventions blended with face-to-face psychotherapy in their depression management. However, they experienced considerable barriers to their referrals, which might have led to their low referral rates in this study. Understanding and addressing their perceived barriers and facilitators might enhance their uptake of bPT as a referral option and therefore improve patients’ access to specialized care.

On the basis of these findings, the following issues should be taken into account when developing an implementation strategy: (1) address the organizational, legal, and reimbursement requirements; (2) consider GPs’ suggestions for implementation, such as the integration of bPT as an additional care pillar in treatment guidelines and the development of measures to increase familiarity with bPT and its advantages (eg, shorter waiting time, improved treatment availability); (3) save GPs’ resources (eg, digital referral receipts, automated feedback about treatment findings, patient leaflet); (4) ensure fit with GPs’ habits and routine activities (eg, interacting with information technology and reimbursement structures in health care); and (5) address GPs’ need for information and training on bPT as well as personal contact and feedback in communication with therapists.

## Appendix

Multimedia Appendix 1 Description of the blended internet-based psychotherapy as intervention and screenshots of the Moodbuster website and app.

Multimedia Appendix 2 Consolidated Criteria for Reporting Qualitative Studies checklist. This table shows the COREQ checklist, a guideline for reporting qualitative studies.

Multimedia Appendix 3 Correlations, means, SDs, and minimum/maximum of general practitioners’ referrals and characteristics (randomized controlled trial; sample N=76). This table shows the correlations between the number of referrals and characteristics, self-ratings on competences, and agreements to statements about depression management.

Multimedia Appendix 4 Descriptive statistics and 95% CI for different general practitioner groups and their mentioned barriers and facilitators (interviewed general practitioners N=12). The contingency tables show the frequency and total numbers of barriers and facilitators in each group (low/high referrers, low/high general practitioner experience, and psychotherapist yes/no).

## Abbreviations

bPT: blended psychotherapy
CBT: cognitive behavioral therapy
E-COMPARED: European Comparative Effectiveness Research on Internet-based Depression Treatment
e–mental health: electronic mental health
GP: general practitioner
IMI: internet- and mobile-based intervention
MDD: major depressive disorder
RCT: randomized controlled trial
TDF: theoretical domains framework
