# Supplementary material for: Barriers and Facilitators for Referrals of Primary Care Patients to Blended Internet-Based Psychotherapy for Depression: Mixed Methods Study of General Practitioners’ Views
Source: JMIR Ment Health. 2020 Aug 18;7(8):e18642. doi: 10.2196/18642 (PMC7463410; doi:10.2196/18642)
Supplement: Multimedia Appendix 1 [file mental_v7i8e18642_app1.docx]

**Multimedia Appendix 1**

**
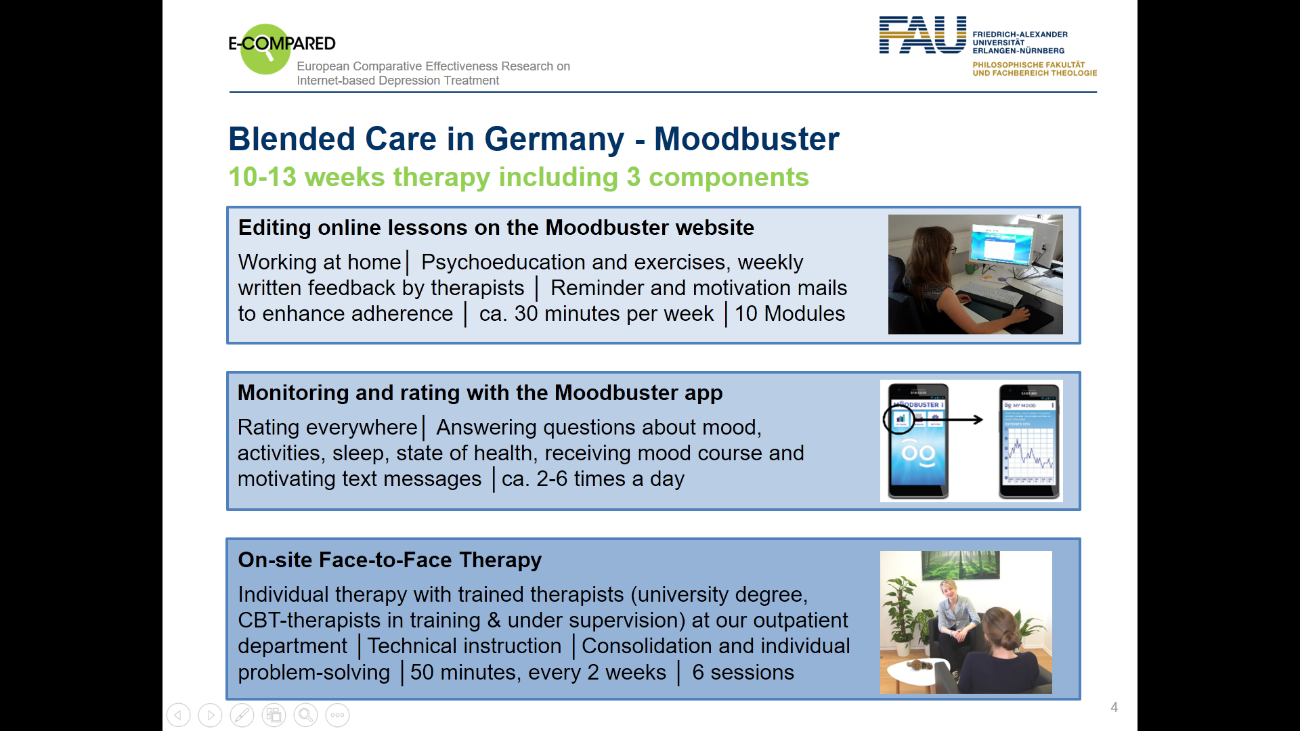
**

Figure 1. Blended internet-based psychotherapy – F2F + Moodbuster Web + App.


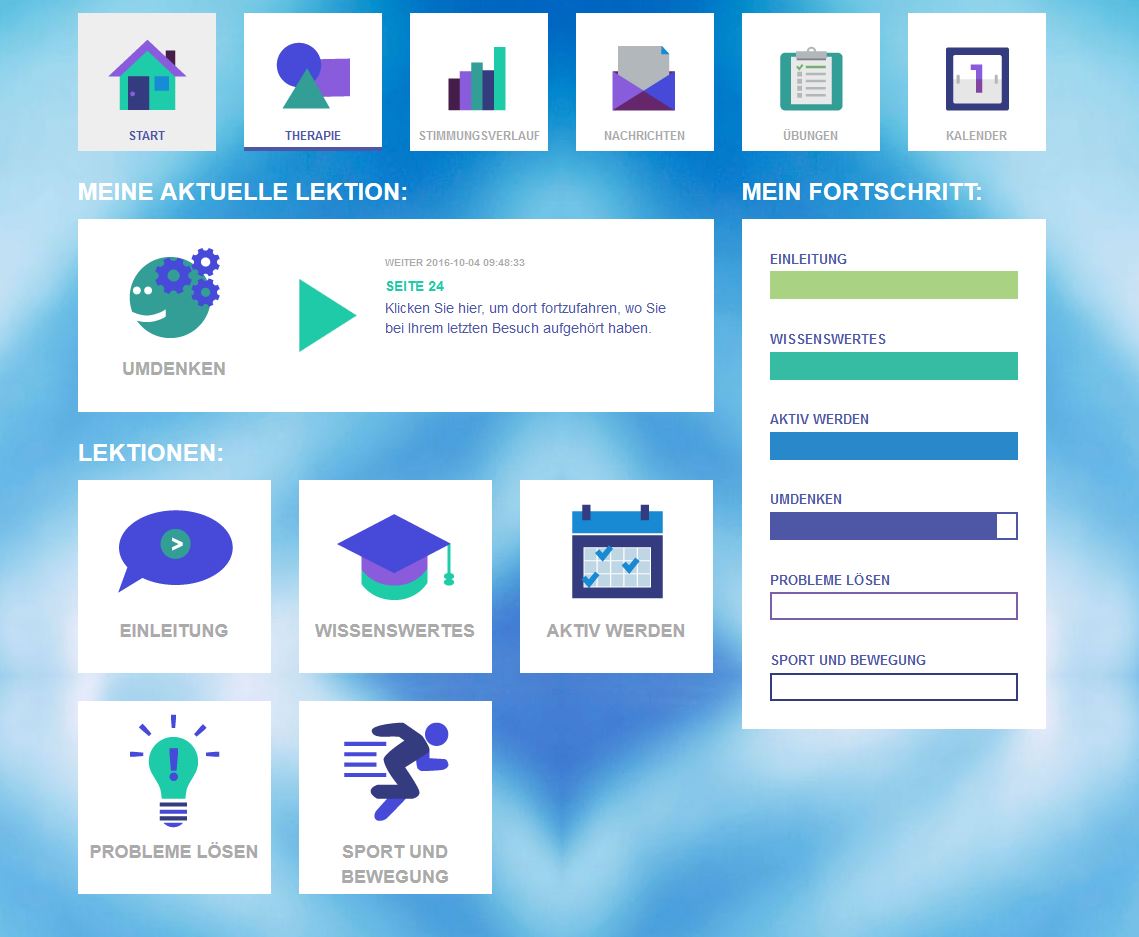


Figure 2. Blended internet-based psychotherapy – Moodbuster Website.


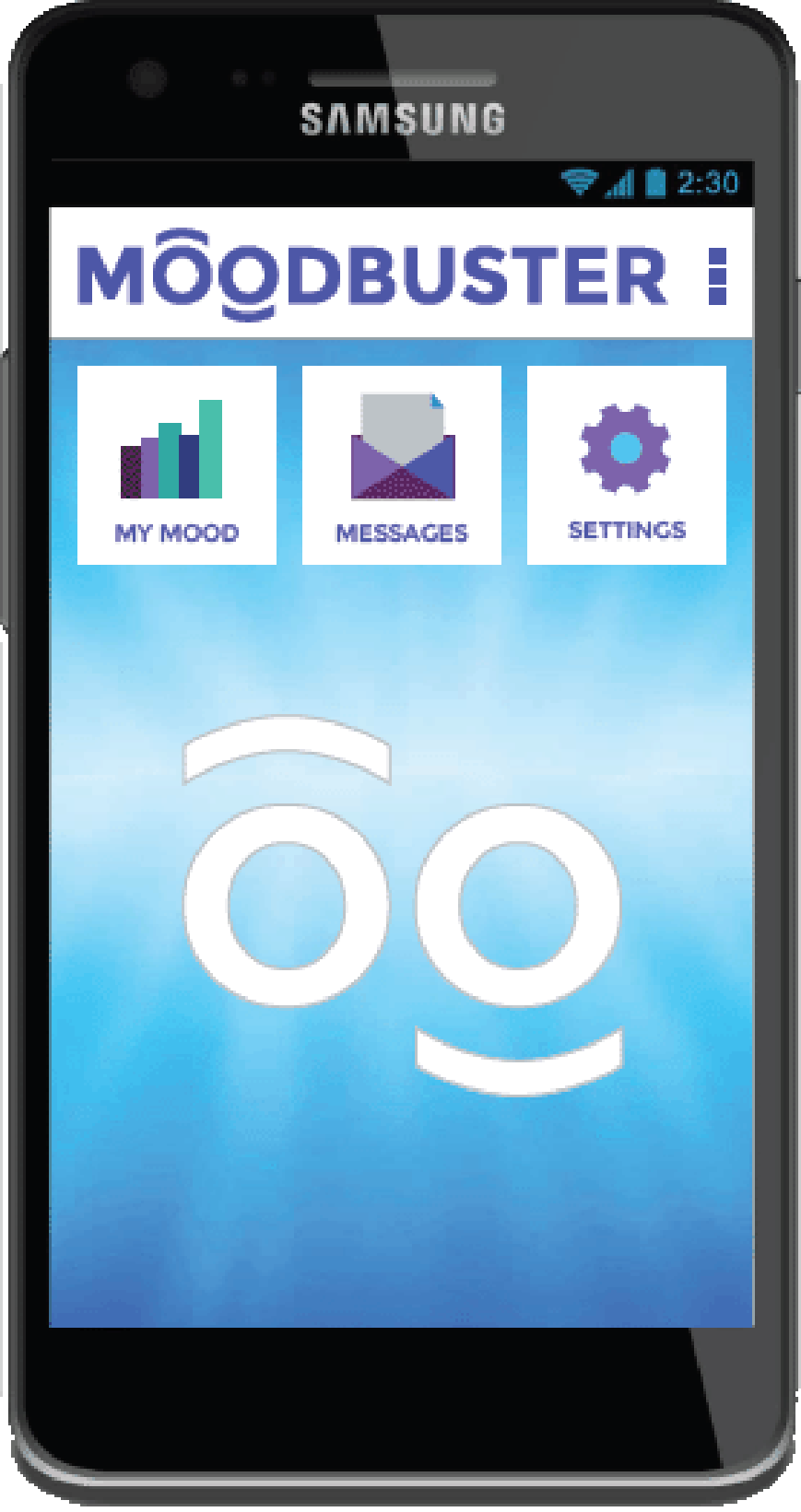

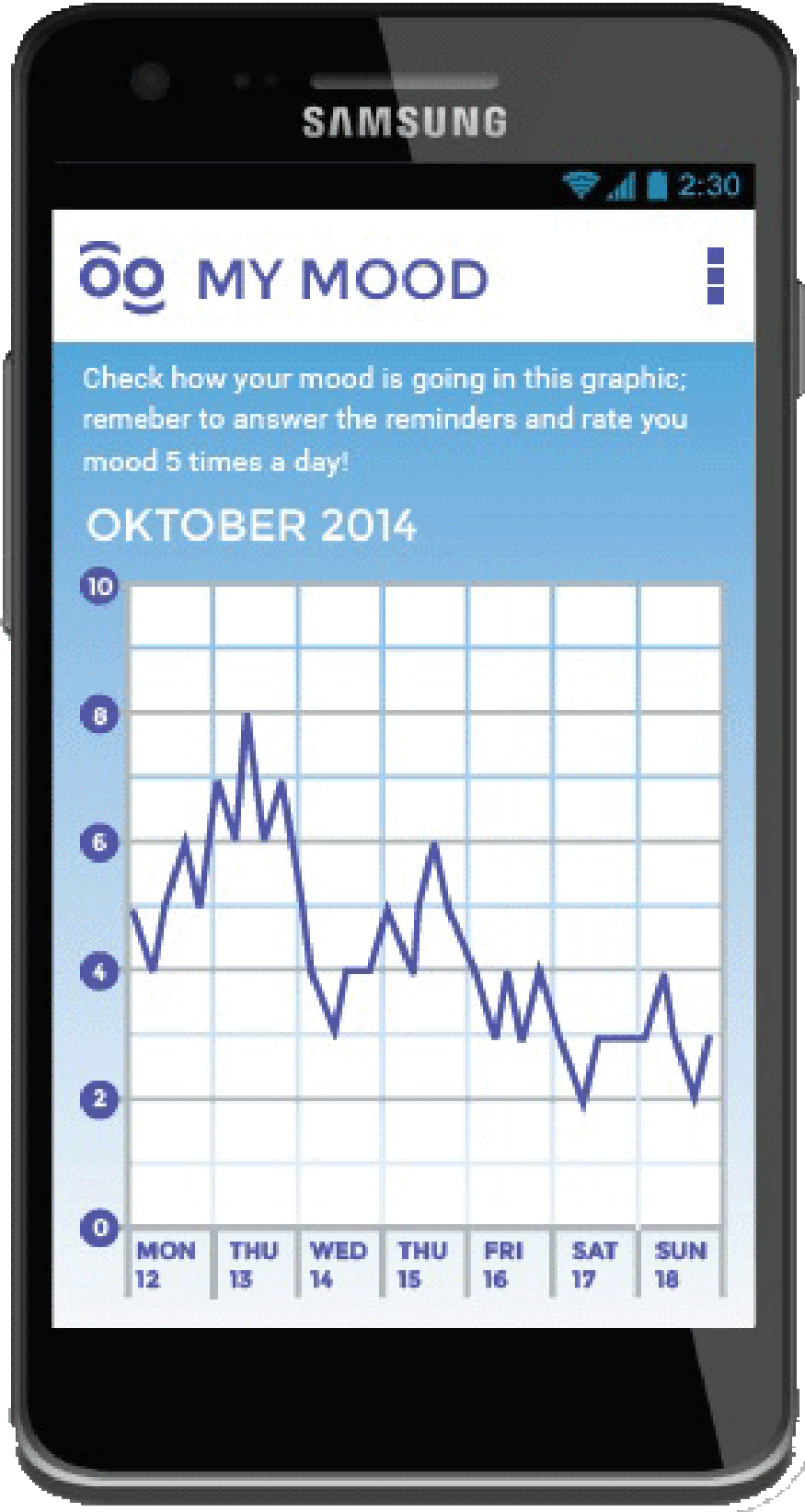

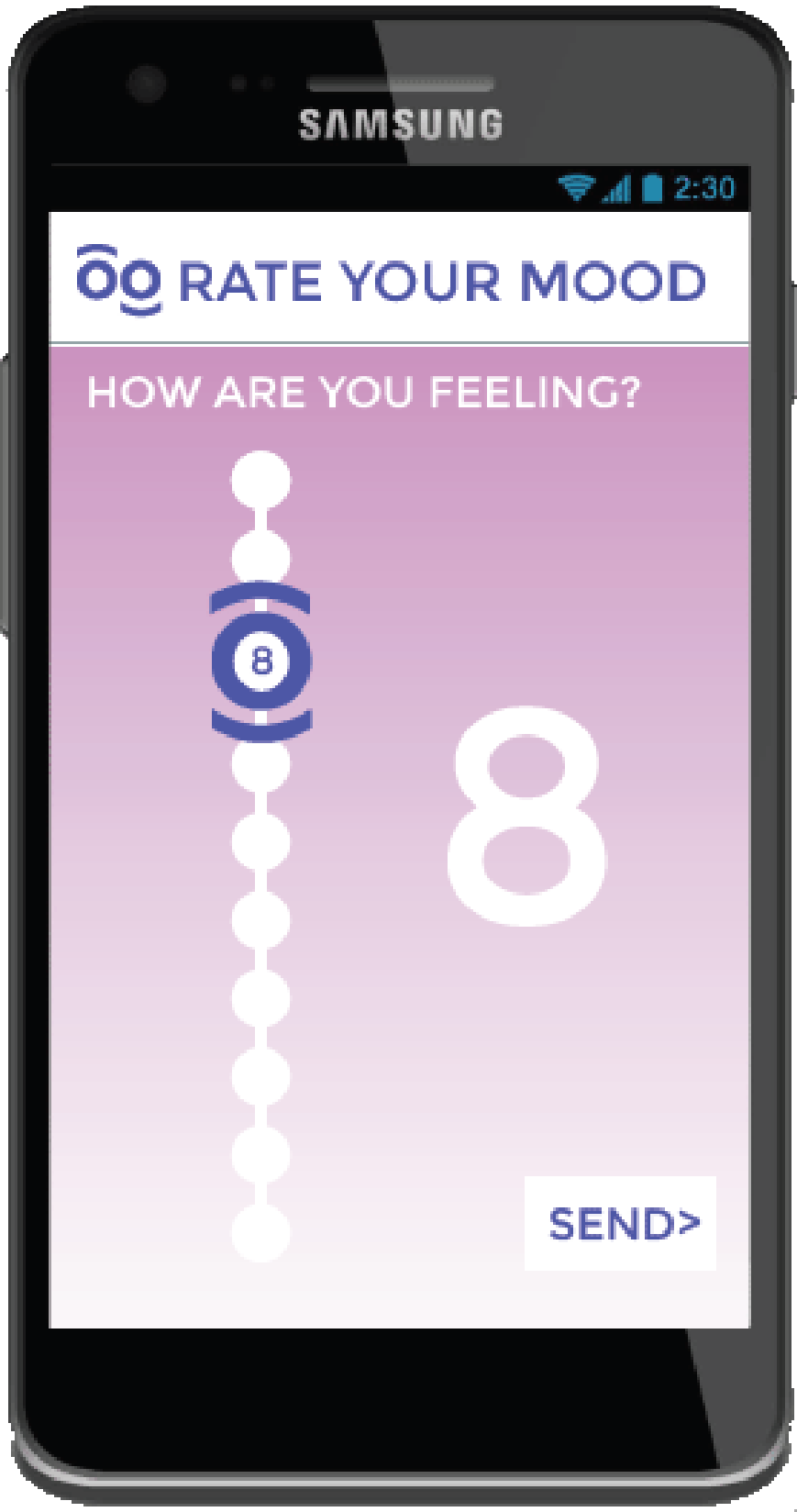


Figure 3. Moodbuster App.


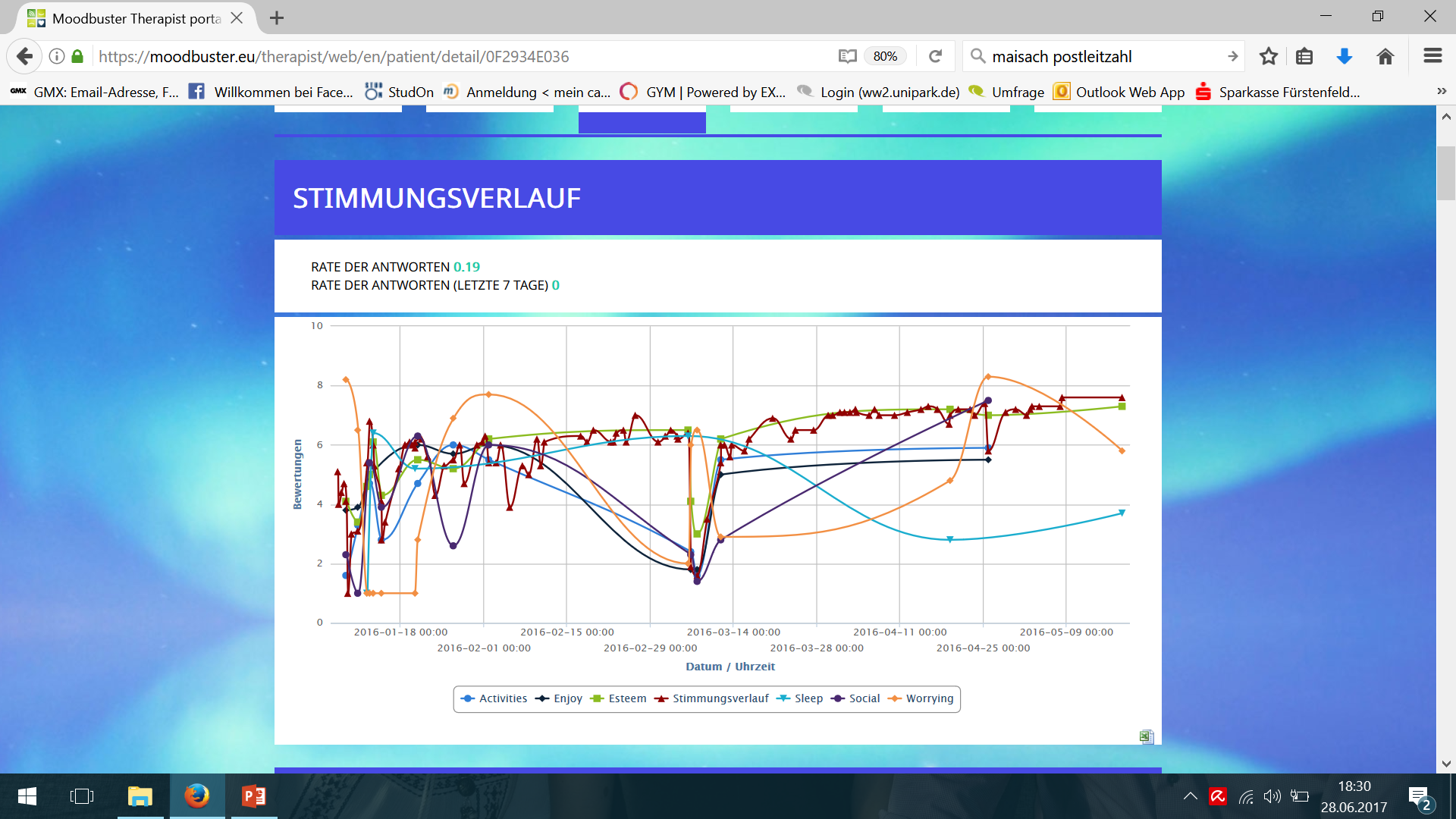


Figure 4. App-based assessments.

Contact: ingrid.titzler@fau.de
