# Supplementary material for: Barriers and Facilitators for Referrals of Primary Care Patients to Blended Internet-Based Psychotherapy for Depression: Mixed Methods Study of General Practitioners’ Views
Source: JMIR Ment Health. 2020 Aug 18;7(8):e18642. doi: 10.2196/18642 (PMC7463410; doi:10.2196/18642)
Supplement: Multimedia Appendix 2 [file mental_v7i8e18642_app2.docx]

| **Multimedia Appendix 2:**  Consolidated criteria for reporting qualitative studies (COREQ): 32-item checklist. | | |
| --- | --- | --- |
| **No. Item** | **Guide questions/description** | **Reported** |
| **Domain 1: Research team and reﬂexivity** | | |
| *Personal Characteristics* | | |
| 1. Interviewer/ facilitator | Which author/s conducted the interviews? | Titzler, Ingrid^1^  Deml, Monika^2^  Wiersma, Caroline^2^ |
| 2. Credentials | What were the researcher’s credentials? E.g. PhD, MD | Master of Science, PhD Student^1^  Bachelor of Science^2^ |
| 3. Occupation | What was their occupation at the time of the study? | Scientific researcher and trial coordinator *E-Compared*^1^  Master thesis at research lab^2^ |
| 4. Gender | Was the researcher male or female? | Female |
| 5. Experience and training | What experience or training did the researcher have? | ^1^ 3 years research experience in health care and treatment; workshop for qualitative studies; experience as clinical therapist  ^2^ Experienced through internship |
| *Relationship with participants* | | |
| 6. Relationship established | Was a relationship established prior to study commencement? | No relationship before start of the E-Compared RCT. Participants cooperated for 1.5 years with the study team before start of the qualitative study. |
| 7. Participant knowledge of the interviewer | What did the participants know about the researcher? e.g. personal goals, reasons for doing the research | The participants were informed about the aims of the involved researcher and the project.  Personal goals: none.  Reasons for doing the expert interviews were known and stated at beginning of the interviews. |
| 8. Interviewer characteristics | What characteristics were reported about the interviewer/facilitator? e.g. Bias, assumptions, reasons and interests in the research topic | Participants were told that the interviewer is interested in implementation science and the aim of the interview was getting insights for later implementation efforts for blended psychotherapy. |

| **Multimedia Appendix 2:**  COREQ (continued) | | |
| --- | --- | --- |
| **No. Item** | **Guide questions/description** | **Reported** |
| **Domain 2: study design** | | |
| *Theoretical framework* | | |
| 9. Methodological orientation and Theory | What methodological orientation was stated to underpin the study? e.g. grounded theory, discourse analysis, content analysis | Theory basis (TDF) of interview guide, content analysis |
| *Participant selection* | | |
| 10. Sampling | How were participants selected? e.g. purposive, convenience, consecutive, snowball | Purposive, GPs were all referral partner of the E-Compared project |
| 11. Method of approach | How were participants approached? e.g. face-to-face, telephone, email | Email, telephone |
| 12. Sample size | How many participants were in the study? | 12 of 110 invited GPs took part |
| 13. Non-participation | How many people refused to participate or dropped out? Reasons? | 39 refused to participate during four reminder activities, others were not reached by telephone and didn’t answered to emails |
| *Setting* |  |  |
| 14. Setting of data collection | Where was the data collected? e.g. home, clinic, workplace | GPs at family practice/workplace (9), at home (2) or at car (1) |
| 15. Presence of non-participants | Was anyone else present besides the participants and researchers? | No |
| 16. Description of sample | What are the important characteristics of the sample? e.g. demographic data, date | Male and female GPs in all age groups with a university degree in medicine with own general practice (see description table) |
| *Data collection* |  |  |
| 17. Interview guide | Were questions, prompts, guides provided by the authors? Was it pilot tested? | Questions, prompts & guides were provided. Pilot testing of interview guide with the first interview (IT). Feedback by IT to the first conducted interviews of MD and CW. |
| 18. Repeat interviews | Where repeat interviews carried out? If yes, how many? | No |
| 19. Audio/visual recording | Did the research use audio or visual recording to collect the data? | Audio recording was used |
| 20. Field notes | Were ﬁeld notes made during and/or after the interview or focus group? | Yes, field notes were made during the interview |
| 21. Duration | What was the duration of the interviews or focus group? | *M* = 56 minutes, *SD* = 15.26, *Min* = 36 minutes, *Max* = 78 minutes |
| 22. Data saturation | Was data saturation discussed? | Yes, it was reached |
| 23. Transcripts returned | Were transcripts returned to parti-cipants for comment/correction? | No |
| **Multimedia Appendix 2:**  COREQ (continued) | | |
| **No. Item** | **Guide questions/description** | **Reported** |
| **Domain 3: analysis and ﬁndings** | | |
| *Data analysis* |  |  |
| 24. Number of data coders | How many data coders coded the data? | 2 |
| 25. Description of the coding tree | Did authors provide a description of the coding tree? | Yes |
| 26. Derivation of themes | Were themes identiﬁed in advance or derived from the data? | Themes were derived from the data as an inductive approach;  themes of the theoretical framework were taken into account |
| 27. Software | What software, if applicable, was used to manage the data? | MAXQDA, Version 12, F4, Audacity |
| 28. Participant checking | Did participants provide feedback on the ﬁndings? | Yes, agreement to identified categories was given |
| *Reporting* |  |  |
| 29. Quotations presented | Were participant quotations presented to illustrate the themes/ﬁndings? Was each quotation identiﬁed (e.g. ID)? | Yes |
| 30. Data and ﬁndings consistent | Was there consistency between the data presented and the ﬁndings? | Yes |
| 31. Clarity of major themes | Were major themes clearly presented in the ﬁndings? | Yes |
| 32. Clarity of minor themes | Is there a description of diverse cases or discussion of minor themes? | Yes |
| Note. Tong A, Sainsbury P, Craig J. Consolidated criteria for reporting qualitative research (COREQ): a 32-item checklist for interviews and focus groups. International Journal for Quality in Health Care. 2007. Volume 19, Number 6: pp. 349 – 357. | | |
